# “Evolution and financial cost of socioeconomic inequalities in ambulatory care sensitive conditions: an ecological study for Portugal, 2000–2014”

**DOI:** 10.1186/s12939-017-0642-7

**Published:** 2017-08-16

**Authors:** Klára Dimitrovová, Cláudia Costa, Paula Santana, Julian Perelman

**Affiliations:** 1 Escola Nacional de Saúde Pública, Universidade NOVA de Lisboa, Avenida Padre Cruz, 1600-560 Lisbon, Portugal; 2 Centre of Studies on Geography and Spatial Planning, University of Coimbra, Faculdade de Letras Colégio de S. Jerónimo, 3004-530 Coimbra, Portugal; 3 Centre of Studies on Geography and Spatial Planning, Department of Geography, University of Coimbra, Faculdade de Letras Colégio de S. Jerónimo, 3004-530 Coimbra, Portugal; 4 Centro de Investigação em Saúde Pública, Escola Nacional de Saúde Pública, Universidade NOVA de Lisboa, Avenida Padre Cruz, 1600-560 Lisbon, Portugal

**Keywords:** Ambulatory care sensitive conditions, Primary care, Socioeconomic inequalities, Costs

## Abstract

**Background:**

Hospitalizations for Ambulatory Care Sensitive Conditions (ACSC) are specific conditions for which hospitalization is thought to be avoidable through patient education, health promotion initiatives, early diagnosis and by appropriate chronic disease management, and have been shown to be greatly influenced by socioeconomic (SE) characteristics. We examined the SE inequalities in hospitalization rates for ACSC in Portugal, their evolution over time (2000–2014), and their associated financial burden.

**Methods:**

We modeled municipality-level ACSC hospitalization rates per 1000 inhabitants and ACSC hospitalization-related costs per inhabitant, for the 2000–2014 period (*n* = 4170), as a function of SE indicators (illiteracy and purchasing power, in quintiles), controlling for the proportion of elderly, sex, disease specific mortality rate, population density, PC supply, and time trend. The evolution of inequalities was measured interacting SE indicators with a time trend. Costs attributable to ACSC related hospitalization inequalities were measured by the predicted values for each quintile of the SE indicators.

**Results:**

Hospitalization rate for ACSC was significantly higher in the 4th quintile of illiteracy compared with the 1st quintile (beta = 1.97; *p* < 0.01), and significantly lower in the 5th quintile of purchasing power, compared with the 1st quintile (beta = − 1.19; *p* < 0.05). ACSC hospitalization-related costs were also significantly higher in the 4th quintile of illiteracy compared with the 1st quintile (beta = 4.04€; *p* < 0.05), and significantly lower in the 5th quintile of purchasing power, compared with the 1st quintile (beta = − 4,69€; *p* < 0.01). The SE gradient significantly increased over the 2000–2014 period, and the annual cost of inequalities were estimated at more than 15 million euros for the Portuguese NHS.

**Conclusion:**

There was an increasing SE patterning in ACSC related hospitalizations, possibly reflecting increasing SE inequalities in early and preventive high-quality care, imposing a substantial financial burden to the Portuguese NHS.

## Background

Hospitalizations for Ambulatory Care Sensitive Conditions (ACSC) have been largely studied as an indirect measure of access to effective Primary Care (PC) [1]. ACSC related hospitalizations are defined as specific conditions (e.g., asthma, angina, heart failure, diabetes and hypertension) for which hospitalization is thought to be avoidable through patient education, health promotion initiatives, early diagnosis, early treatment, and by appropriate chronic disease management, i.e., by “timely and effective PC” [2, 3]. The reason for considering these conditions as avoidable is that they can be managed in order to avoid their clinical progression. For example, early diagnosis, adequate treatment and change in lifestyle should avoid the hospitalization of a person with diabetes.

Despite evidence that increased physician supply and greater diffusion of PC are associated with lower rates of ACSC hospitalizations [4, 5], many authors suggest that ACSC related hospitalizations are more closely related to socioeconomic (SE) characteristics than with the quality of PC [6, 7]. Many studies have indeed found a strong SE gradient in the rates of hospitalizations for ACSC, showing that people from low-income areas, people living in rural and/or more deprived areas, and people from areas with higher proportions of uneducated people have a much higher risk of being hospitalized for these conditions [3, 8–10]. These SE disparities have been described not only in countries without universal health coverage, like the US, but were also found in countries like Canada [11], Italy [12], the United Kingdom [13], and Sweden [14], where there are little financial barriers in access to PC.

We can classify explanations for SE inequalities in ACSC related hospitalizations into two categories: those related to individual characteristics and those related to contextual characteristics. With regard to individual characteristics, patients with lower literacy may be less prone to adopt self-management behaviors [13]; they may have a lower probability of enrolling in health promotion activities [15]; they may have a poor understanding of physicians’ recommendations, leading to poorer treatment compliance [9]; they may lack awareness and knowledge of the health care system [15]; and they may face language and cultural barriers [16]. Patients with low income may also delay the search for needed medical care or medications due to financial constraints on transportation costs and healthcare co-payments [17].

Regarding contextual factors, authors suggest that patients from lower-income areas may face a shortage of PC physicians and fewer referrals to specialist consultations [13, 18], leading to a lower continuity of care and to a delay in care [19]. We can hypothesize also that the quality of services is lower in more deprived and rural areas, due for example to insufficient financing. In addition, more individuals in low-income areas are hospitalized for social reasons, due to non-response or lack of coordination with social services [18]. Finally, access to PC may be influenced in SE disadvantaged areas through the availability of transportation systems and by the distance to PC facilities [7]. Most studies on this topic are ecological, and authors use SE characteristics of the area of residence of the individual to explore these associations, since it is believed that the aggregate SE characteristics reflect the nature of the social environment where people live [20].

In this paper we measured SE inequalities in ACSC related hospitalizations, their evolution over time, and financial consequences for the Portuguese National Health Service (NHS). The use of a long period of time permits a more consistent measurement of inequalities, and testing whether these SE inequalities in ACCS related hospitalizations have risen, potentially contributing to the growing relative inequalities in health in the last decade [21]. Finally, by measuring the costs attributable to inequalities, we estimate the potential savings that would be achieved and potentially reallocated to other needs.

The case of Portugal is of interest because it combines a universal coverage in PC, characterized by relatively low co-payment rates and a large network of PC practices, with a relatively weak social welfare system. Portugal is also one of the EU countries with the highest inequalities in income distribution and risk of poverty, [22] and recent evidence showed considerable SE health inequalities [23] and strong associations between material deprivation and mortality [24].

## Methods

### Data sources

We used data from the Portuguese Central Administration of the Health System on all in-patient stays at all public non-specialized Portuguese NHS hospitals for the years 2000 to 2014 (*n* = 11,129,000). This database includes, for each patient, several clinical (e.g. diagnosis, comorbidities) and demographic characteristics (e.g. age, sex, residence geographic code). Using information from the patients’ residence geographic code, the data on ACSC related hospitalizations were aggregated by municipality and year, and merged with municipality and year-level data on demographic, SE, and PC physician supply data from the National Institute for Statistics. Our total number of observations was *n* = 4170, which corresponds to the total number of municipalities (*n* = 278) followed over the 15-years period. Note that the municipality is the second lowest administrative level in Continental Portugal.

### Dependent variable

We used the set of codes for ACSC related hospitalizations as defined by the US Agency for Healthcare Research and Quality (AHRQ), which are called Prevention Quality Indicators (PQIs). We opted for the AHRQ definition because it is widely used in the US, and has also been adapted and adopted by different European countries including Spain [25] and Italy [26], allowing for the comparison of results. We also used this approach because it is in accordance to the Portuguese in-patient disease classification system (ICD-9-CM). We used the overall set of 12 PQIs validated for adult population, defined as “PQI 90 Overall composite” (Table 1). The detailed list of inclusion and exclusion of ICD-9-CM codes for the construction of PQIs is described elsewhere [27].

**Table 1:** Ambulatory care sensitive conditions (Prevention Quality Indicators) defined by Agency for Healthcare Research and Quality

Individual Prevention Quality Indicators (PQIs)	
PQI 01 Diabetes Short-term Complications Admissions Rate	
PQI 03 Diabetes Long-term Complications Admission Rate	
PQI 05 COPD or Asthma in Older Adults Admission Rate	
PQI 07 Hypertension Admission Rate	
PQI 08 Heart Failure Admission Rate	
PQI 10 Dehydration Admission Rate	
PQI 11 Bacterial Pneumonia Admission Rate	
PQI 12 Urinary Tract Infection Admission Rate	
PQI 13 Angina without Procedure Admission Rate	
PQI 14 Uncontrolled Diabetes Admission Rate	
PQI 15 Asthma in Younger Adults Admission Rate	
PQI 16 Rate of Lower-Extremity Amputation Diabetes	
Composite Prevention Quality Indicators (PQIs)	
PQI 90 Overall composite (includes 01, 03, 05, 07, 08, 10, 11,12, 13, 14, 15, and 16)	

Source: (AHRQ 2015)

Based on the patients’ residence geographic code, we calculated the total number of ACSC related hospitalizations (“PQI 90 Overall composite”) per municipality and year. Then we calculated the rate of ACSC related hospitalizations per 1000 adult inhabitants (≥18 years old) by dividing the total number of ACSC related hospitalizations by the total adult population in the municipality and year. Additionally, we calculated the ACSC-related costs per inhabitant, for each municipality and year. This cost was estimated by computing the total costs of all ACSC related hospitalizations previously identified per municipality and year, using the official Portuguese NHS prices attributed to each in-patient stay, based on its Diagnosis Related Groups (DRG), and then divided by the total adult population in each municipality and year. We used the decree-laws, published regularly by the Ministry of Health, with the official NHS prices which were in force in the period under analysis. [28]. Note that these costs correspond only to the in-patient episode and do not include any other pre-existing health expenses that the patient may have had in other healthcare settings.

### Explanatory variables

We first used as an explanatory variable the municipality year-level purchasing power, which indicates the relative purchasing power in buying goods and services in a given municipality for the average wage in that same municipality. The national purchasing power was used as reference, with a value of 100. That is, if in a given municipality the purchasing power is 60, the inhabitants of this municipality with an average salary can afford 40% fewer typical goods and services than the average residents in Portugal. This indicator is calculated bi-annually; for the in-between years we used average values. Second, we used the municipality-year-level illiteracy rate, defined as the proportion of people aged 10 or more years old who cannot read or write. As mentioned above, illiteracy greatly influences disease prevention and self-management behaviors, leading to a higher risk of hospitalization [15]. In Portugal, illiteracy rates are available only for census years (2001 and 2011), and we therefore assumed a linear trend of the illiteracy rate for the years in-between, and adopted the 2011 values for the subsequent years.

### Covariates

We controlled for the municipality year-level proportion of elderly, sex, disease specific mortality rate, population density and PC supply. The proportion of elderly was measured as the proportion of people with 65 or more years of age in each municipality, since ACSC related hospitalizations are mostly prevalent at older ages, especially after the age of 65 years [13], and sex was measured as the proportion of males in each municipality. Disease-specific mortality rate for diabetes, COPD and ischemic heart diseases (as defined in the European Shortlist for Causes of Death [29]) were used as covariates to adjust for disease prevalence and severity, since areas with higher mortality are more likely to have greater health needs, associated with higher ACSC related hospitalizations [5]. We used these chronic conditions since they are the ones that contribute notably for ACSC related hospitalizations [5]. Population density was used to account for rurality, and PC supply was measured by the rate of PC physicians working in PC practices per 1000 inhabitants in each municipality and year. This indicator is one of the best proxies for PC supply, and has been used by other authors [4]. Finally, we included a time trend, allowing control for the other growth factors in the rate of ACSC related hospitalizations.

### Statistical analysis

We used quintiles of the distribution for our SE explanatory variables, with the first quintile being the lowest. This approach was used by other authors in similar studies [6] and it seeks to obtain categorical values in order to explore nonlinearities. We added all covariates as continuous. Note that for illiteracy the first quintile is the least deprived (less percentage of people with illiteracy), while for purchasing power the first quintile is the most deprived.

We first calculated the absolute difference and the population-attributable risk of the rate of ACSC related hospitalizations per 1000 inhabitants for each SE indicator. The absolute difference aims to measure the difference in ACSC related hospitalizations between opposite quintiles (most deprived versus least deprived). The population-attributable risk corresponds to the difference between the mean rate of ACSC related hospitalizations and the rate of ACSC related hospitalizations of the least deprived quintile, given as a percentage of the mean rate of the ACSC related hospitalizations. In other words, the population-attributable risk describes the reduction in the rate of ACSC related hospitalizations that would be observed if the entire population had the characteristics of the least deprived SE quintile [30].

We then modeled the ACSC hospitalization rates (Model I) and the ACSC-related costs per inhabitant (Model II) as a function of year-specific quintiles for the SE indicators, controlling for the proportion of elderly, sex, disease specific mortality rate, population density, PC supply, and for the time trend. In order to estimate the evolution of SE inequalities in the 2000–2014 period, we interacted the illiteracy rate and purchasing power with the time trend (Model III and IV, respectively), controlling for all the covariates. All four models used longitudinal data analysis (panel data) and were regressed using the ordinary least squares with municipality fixed effects [31]. We used municipality fixed effects since we are interested in analyzing the impact of only the variables that vary over time, and we aim to explore the causes of changes within the entity, i.e., the relationship between explanatory and outcome variables within the municipality. Fixed effects control for all-time invariant differences between the municipalities, so the estimated coefficients of the fixed-effects models cannot be biased due to omitted time-invariant characteristics such as culture, historical determinants, or institutional settings [32]. Additionally, a Hausman test (*p* < 0.01) and a Prob F test (*p* < 0.01) confirmed the utilization of fixed instead of random effects.

Finally, we calculated the costs attributable to ACSC related hospitalization inequalities, from the Portuguese NHS perspective (i.e. the additional direct costs incurred in ACSC related hospitalizations arising as a result of SE inequalities), for the year 2014. With this analysis, we aimed at estimating the total hypothetical savings that would be obtained if the pattern of cost was similar for all quintiles of SE variables. To do so, we first calculated the predicted values for each quintile of the SE indicators of the Model II, in order to estimate the predicted cost per inhabitant by quintile. These predicted values are a post estimation, which means that they are calculated from the predictions of a previously fitted model, in which some of or all the covariates are fixed, for example at their means [33]. We then multiplied the predicted ACSC-related cost per inhabitant by the total adult population in each quintile of illiteracy and purchasing power. We also estimated a hypothetical cost by multiplying the predicted ACSC-related cost per inhabitant of the least deprived quintiles by the total adult population. Lastly, the costs attributable to inequalities were estimated by the difference of the two costs. All the analyses were conducted with Stata version 13 (StataCorp LP, College Station, TX, USA).

## Results

### Descriptive analysis

**Fig. 1:**
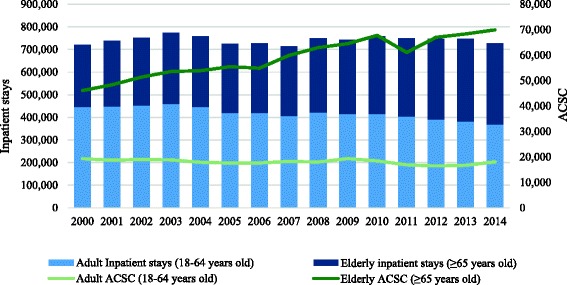
Adult and elderly inpatient stays and hospitalizations for ambulatory care sensitive conditions in Portugal, 2000–2014

In Portugal, between 2000 and 2014, there were on average 741,933 public hospital discharges of adults (age ≥ 18 years) annually, among which 77,077 (10.4%) were potentially avoidable. While the total number of in-patient stays maintained relatively stable over the 2000–2014 period (from 720,414 in 2000 to 727,495 in 2014), the total number of ACSC related hospitalizations increased from 65,401 to 88,006 (a 34.6% increase). Considering only the elderly (age ≥ 65 years), there was a 51.7% increase in ACSC elderly related hospitalizations, which represented 70% and 79% of the total ACSC related hospitalizations in 2000 and 2014, respectively (Fig. 1).

The crude national rate of ACSC related hospitalizations per 1000 inhabitants increased from 8.29 in 2000 to 10.81 in 2014, while the hospital utilization rate decreased from 91.28 per 1000 inhabitants to 89.32 per 1000 inhabitants over the same time period. Table 2 shows the overall descriptive characteristics of our unit of analysis (municipalities) in 2000 and 2014.

**Table 2:** Sample characteristics: ambulatory care sensitive conditions and their determinants in Portugal, 2000–2014

	2000 (*n* = 278)				2014 (*n* = 278)			
Indicator (per municipality)	Mean	SD	Min	Max	Mean	SD	Min	Max
Rate of ACSC hospitalizations per 1000 inhabitants	8.59	3.31	0.18	22.00	12.55	4.65	4.67	28.15
Total population	35,415	56,851	1918	563,475	35,503	56,926	1739	509,312
Adult population (≥ 18 years old)	28,391	46,286	1597	480,710	29,297	46,549	1457	422,951
Elderly population (≥ 65 years old)	5839	9742	498	132,637	7314	12,069	391	141,742
Proportion of elderly (≥ 65 years) (%)	21.08	6.59	8.42	40.84	24.37	6.09	11.81	44.97
Proportion of males (%)	47.85	1.18	43.10	53.01	47.01	1.07	43.12	52.48
Proportion of illiterate people (%)	14.04	5.72	3.90	33.25	6.76	3.32	1.75	17.57
Purchasing power	66.65	28.91	33.72	305.19	82.40	17.26	57.89	201.75
Primary care physicians per 1000 inhabitants	0.76	0.20	0.17	1.79	0.71	0.21	0.00	1.44
Diabetes mortality rate per 1000 inhabitants	0.46	0.27	0.00	1.46	0.69	0.39	0.00	2.77
COPD mortality rate per 1000 inhabitants	0.95	0.52	0.00	3.49	1.22	0.82	0.00	6.22
Heart disease mortality rate per 1000 inhabitants	1.25	0.82	0.14	7.11	1.01	0.56	0.22	4.32
Population density (N.°/km^2^)	307.00	870.11	6.60	7517.70	303.90	830.88	4.40	7397.70

Source: authors

### Univariate analysis

**Fig. 2:**
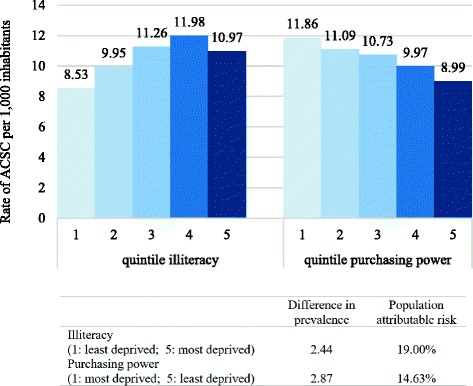
Rate of hospitalizations for ambulatory care sensitive conditions by socioeconomic quintiles in Portugal, 2000–2014

The absolute difference is of 2.44 ACSC related hospitalizations per 1000 inhabitants for illiteracy and 2.87 ACSC related hospitalizations per 1000 inhabitants for purchasing power (Fig. 2). If we could decrease the illiteracy levels to the lowest quintile, we would reduce the rate of ACSC related hospitalizations by 19.00% (population-attributable risk). Similarly, if we could increase the purchasing power to the highest quintile, we would reduce the rate of ACSC related hospitalizations by 14.63%.

### Multivariate analysis

The rate of ACSC related hospitalizations was positively and significantly associated with illiteracy, being 1.32 (*p* < 0.01), 1.10 (*p* < 0.05), 1.97 (*p* < 0.01), and 1.59 (*p* = 0.05) higher for quintiles two, three, four, and five, respectively in comparison to the first quintile (Model I) (Table 3). Similarly, the ACSC-related costs per inhabitant were 2.37€ (*p* < 0.05) and 4.04€ (*p* < 0.05) higher for the second and fourth quintile of illiteracy in comparison to the first quintile (Model II) (Table 3). The rate of ACSC related hospitalizations was significantly and negatively associated with purchasing power, being 0.86 (*p* < 0.01), 1.21 (*p* < 0.01), and 1.19 (*p* < 0.01) lower for the third, fourth, and fifth quintiles, respectively, in comparison to the first quintile (Model I) (Table 3). The ACSC-related costs per inhabitant were also significantly lower, being 3.07€ (*p* < 0.01), 4.34€ (*p* < 0.01), and 4.69€ (*p* < 0.01) lower for the third, fourth and fifth quintiles of purchasing power respectively, in comparison with the first quintile (Model II) (Table 3).

**Table 3:** Association between socioeconomic factors with the rate of ACSC and ACSC related-costs in Portugal, 2000–2014

		MODEL I		MODEL II	
		Rate of ACSC		ACSC related costs	
		β	(95% CI)	β	(95% CI)
Trend		0,23***	(0,19; 0,27)	0,53***	(0,43; 0,63)
SE indicators					
Illiteracy	1 (least deprived)				
	2	1,32***	(0,59; 2,08)	2,37**	(0,28; 4,47)
	3	1,10**	(0,04; 2,16)	2,47*	(−0,46; 5,40)
	4	1.97***	(0,59; 3,34)	4,04**	(0.24; 7.83)
	5 (most deprived)	1,59*	(−0.01; 3,18)	2.47	(−1.94; 6.88)
Purchasing power	1 (most deprived)				
	2	0.00	(−0,46; 0,46)	−0.56	(−1,83; 0,72)
	3	−0.86***	(−1,46; −0,27)	−3,07***	(−4.71; −1,43)
	4	−1,21***	(−1,92; −0,50)	−4,34***	(−6,30; −2,39)
	5 (least deprived)	−1,19***	(−2,08; −0,30)	−4,69***	(−7,15; −2,22)
Confounders					
Proportion of elderly (≥ 65 years)		0.04	(−0.06; 0.15)	0.23	(−0.07; 0.52)
Proportion of males		−0.01	(−0.33; 0.31)	0.80*	(−0.09; 1.68)
Diabetes mortality rate per 1000 inhabitants		0.26*	(−0.03; 0.54)	1.07***	(0.29; 1.86)
COPD mortality rate per 1000 inhabitants		1.01***	(0.83; 1.20)	2.61***	(2.10; 3.11)
Heart disease mortality rate per 1000 inhabitants		0.33***	(0.12; 0.53)	1.23***	(0.66; 1.80)
Population density (N.°/km^2^)		−0.00	(−0.00; 0.00)	−0.00	(−0.01; 0.00)
Primary care physicians per 1000 inhabitants		−1.29***	(−1.97; −0.60)	−3.08***	(−4.97; −1.19)

***p < 0.01; **p < 0.05; *p < 0.1

R^2^ overall of MODEL I = 0.14

R^2^ overall of MODEL II = 0.12

Furthermore, the rate of ACSC related hospitalizations was positively and significantly associated with the COPD mortality rate and with the heart disease mortality rate. For each unit increase in the COPD mortality rate and for each unit increase in the heart disease mortality rate, there is an increase of 1.01 (*p* < 0.01) and 0.33 (*p* < 0.01) hospitalizations for ACSC per 1000 inhabitants. The ACSC-related costs per inhabitant were also significantly higher for these indicators, as well as for the diabetes mortality rate. Regarding PC supply, each unit increase in the rate of PC physicians per 1000 inhabitants was associated with a decrease of 1.29 (*p* < 0.01) hospitalizations for ACSC per 1000 inhabitants and with a decrease of 3.08€ (*p* < 0.01) ACSC-related costs per inhabitant. In our study, the proportion of elderly, proportion of males and population density were not statistically significantly associated with the rate of ACSC related hospitalizations and with the ACSC-related costs. Finally, the time trend shows that overall the rate of ACSC related hospitalizations and the ACSC-related costs per inhabitant increased in the 2000–2014 period. Each year there was an increase of 0.23 (*p* < 0.01) in the rate of ACSC related hospitalizations, and an increase of 0.53€ (*p* < 0.01) in ACSC-related costs per inhabitant.

The interaction between the time trend and illiteracy was positive and significant for the rate of ACSC related hospitalizations while the interaction between the time trend purchasing power was negative and significant (Model III) (Table 4), indicating in both cases an increase in SE inequalities. Specifically, each year there was a statistical significant increase in the rate of ACSC related hospitalizations of 0.42 (*p* < 0.01), 0.41 (*p* < 0.01), and 0.37 (*p* < 0.01) for the third, fourth, and fifth quintiles of illiteracy, respectively, in comparison to the first quintile. Similarly, there was a statistical significant decrease in the rate of ACSC related hospitalizations of 0.16 (*p* < 0.01), 0.10 (*p* < 0.01), 0.14 (*p* < 0.01) and 0.05 (*p* < 0.01) for the second, third, fourth, and fifth quintiles of purchasing power, respectively, in comparison to the first quintile. The interactions had similar results for ACSC-related costs per inhabitant (Model IV) (Table 4).

**Table 4:** Interaction between time trend and socioeconomic indicators with ACSC and ACSC related-costs in Portugal, 2000–2014

		MODEL III		MODEL IV	
		Rate of ACSC		ACSC related costs	
		β	(95% CI)	β	(95% CI)
Trend		0,28***	(0,19; 0,38)	0.68***	(0,42; 0.94)
SE indicators					
Illiteracy#trend	1 (least deprived)				
	2	0,00	(−0,06; 0,07)	−0,02	(−0,19; 0,15)
	3	0,14***	(0,07; 0,20)	0,32***	(0,13; 0,51)
	4	0,13***	(0,05; 0,21)	0,27**	(0,05; 0,48)
	5 (most deprived)	0,09**	(0,01; 0,17)	0,21*	(−0.01; 0,43)
Purchasing power#trend	1 (most deprived)				
	2	−0,12***	(−0,19; −0,05)	−0,26***	(−0,44; −0,07)
	3	−0,18***	(−0,25; −0,12)	−0,47***	(−0,66; −0,29)
	4	−0,14***	(−0,22; −0,07)	−0,42***	(−0,62; −0,22)
	5 (least deprived)	−0,23***	(−0,31; −0,15)	−0,49***	(−0,71; −0,27)

***p < 0.01; **p < 0.05; *p < 0.1

Note: All values were adjusted for illiteracy, purchasing power, proportion of elderly, proportion of males, diabetes mortality rate per 1000 inhabitants, COPD mortality rate per 1000 inhabitants, heart disease mortality rate per 1000 inhabitants, population density and PC supply. We exclude these variables from the table to simplify the reading

### Inequality-related costs

In order to estimate the costs attributable to ACSC related hospitalizations inequalities, we first calculated the predicted values (predicted cost per inhabitant in each quintile of the SE indicators) for the previously fitted Model II (Table 5). All the other covariates were set at their mean values, and the estimation was not set as balanced in order to take into account the unequal sample size in each quintile [33]. We then multiplied the total adult population in each quintile by the corresponding predicted cost per inhabitant, and obtained a total cost of 207,803,901€ for illiteracy, and 205,661,714€ for purchasing power. Then we calculated a hypothetical cost by multiplying the total adult population in the year 2014 (*n* = 8,144,649) by the predicted cost of the least deprived quintiles of illiteracy (24.49€) and purchasing power (24.35€), assuming that all municipalities behaved as the least deprived SE quintiles (199,483,712€ for illiteracy and 198,342,076€ for purchasing power). The costs attributable to inequalities were then estimated by the difference between the “real” and the hypothetical costs, i.e. 8,320,190€ for illiteracy and 7,319,638€ for purchasing power. Therefore, if all municipalities behaved as the least deprived SE quintiles, the Portuguese NHS would save 15,639,828€ per year.

**Table 5:** Predicted values for ambulatory care sensitive conditions related costs, by socioeconomic indicators, in Portugal, 2000–2014

ACSC related costs	Quintile	Predicted values (€)	(95% CI)	Adult population by quintile in 2014	Cost (€)
Illiteracy	1 (least deprived)	24.49***	(22.20; 26.79	4,751,169	116,368,529
	2	26.77***	(25.15; 28.38)	1,610,426	43,106,659
	3	26.53***	(25.15; 27.92)	922,640	24,482,234
	4	28.41***	(26.58; 30.24)	493,458	14,020,667
	5 (most deprived)	26.78***	(24.25; 29.31)	366,956	9,825,812
Total				8,144,649	207,803,901
Purchasing power	1 (most deprived)	29.19***	(27.87; 30.52)	374,834	10,943,140
	2	28.67***	(27.75; 29.60)	625,682	17,940,993
	3	26.13***	(25.34; 26.93)	1,033,524	27,011,139
	4	24.82***	(23.93; 25.71)	2,048,886	50,853,576
	5 (least deprived)	24.35***	(22.91; 25.79)	4,061,723	98,912,866
Total				8,144,649	205,661,714

***p < 0.01

Note: All other variables of the regression (as used in Model II) were set at their means for the estimation of the predicted values for each SE indicator

## Discussion

### Key findings and interpretation

First, our data show that the national crude rate of ACSC related hospitalizations increased from 8.29 to 10.81 per 1000 inhabitants between 2000 and 2014. These overall rates are very close to those observed in other European countries, such as Italy [26] and France [34].

Second, our study highlights significant SE disparities in ACSC related hospitalizations. The most disadvantaged municipalities, i.e., those with the highest levels of illiteracy and lowest levels of purchasing power, had the highest rates of ACSC related hospitalizations. In the univariate analysis, we estimated a reduction of 19.00% and 14.63% (population-attributable risk) in ACSC related hospitalization rates, if all municipalities had the illiteracy levels and purchasing power of the municipalities from the least deprived quintiles. These inequalities were largely confirmed in the multivariate analysis (i.e. adjusted for the proportion of elderly, proportion of males, diabetes, COPD and heart disease mortality rate per 1000 inhabitants, population density and for PC supply), and were consistent with earlier research performed in other countries with National Health Services. For Italy, authors found a risk ratio of 2.59 (95% CI: 2.35–2.85) for ACSC related hospitalizations, between the highest and lowest quintile of area income [12]. For Ireland, an incidence rate ratio of 4.29 (95% CI 4.20–4.39) between opposite quintiles of a deprivation index was found in the rate of ACSC related hospitalizations between 2002 and 2013 [35]. Also, for Canada, authors found that patients in the lowest area income quintiles were approximately three times as likely to be hospitalized for an ACSC, relative to their counterparts in the highest area income quintile (odds ratio = 2.93, 95% CI 2.19–3.93) [11]. Another study for Canada that focused on disparities in preventable heart attack hospitalizations showed that the hospitalization rate would be 16% lower if rates for all SE groups matched those in the areas with the highest income [36].

There are individual and contextual factors that may contribute to the SE inequalities in ACSC related hospitalizations. Since our study was ecological, we relied on area-based SE variables. However, these variables can reflect both individual and contextual effects. On the one hand, area-based SE factors may proxy individual SE characteristics, that is, people living in more deprived areas are more likely to have lower literacy levels and to experience material and financial deprivation. Therefore, they are less likely to adopt self-management behaviors since they have a poorer understanding of how the disease affects their life, of how to cope with the symptoms, and of how to maintain good control throughout the course of the disease [13]; and they are more likely to experience delay in care due to transportation costs and due to lack of knowledge of the health care system. On the other hand, area-based SE factors can reflect the SE context where people live [20]. In lower-income areas there may be lower cultural and social support for early care and prevention, and the access to PC may be affected by the distance to PC facilities and by the availability of transportation systems.

Third, we observe a widening of SE inequalities in the rate of ACSC related hospitalizations over the 15-year period examined. To the best of our knowledge, no other study focusing on SE inequalities and ACSC related hospitalizations has reported the evolution of inequalities over time. Nevertheless, relative SE inequalities in health in Europe have not only persisted in the last three decades but also widened, according to recent measures [21, 37]. Our findings are consistent with these, suggesting that increasing inequalities in ACSC related hospitalizations, which reflect inequalities in early and preventive care, are a possible explanation for these widening disparities in health.

Finally, we estimate potential savings of more than 15 million euros per year from reducing inequalities, for the Portuguese NHS. It is worth noting that these estimates aimed only to provide an indication of the likely scale of the costs of health inequalities, since it is hypothetical to assume that everyone would have the same illiteracy levels and purchasing power as the 20% of the least deprived population. Also, not all differences observed in the distribution of the rate of ACSC related hospitalizations result from SE inequalities. There will always be differences in the distribution of illness and disease among people, regardless of their SE status [38]. Moreover, the reduction of inequalities in ACSC related hospitalizations would certainly require substantial investments, so our values for potential in-patient savings provide just a benchmark to which these investments can be compared. Despite the limitations of these assumptions, other studies use this approach [38, 39].

### Limitations

There are limitations to our study that must be addressed. First, even though most countries use a list of ACSC codes based on the initial definition from the US, different sets of ACSC codes may result in different estimation rates. Some authors suggest that the choice of ACSC related hospitalizations should be country specific due to variations in practices and health systems among countries [2], but other authors believe that access barriers to care, especially for the most disadvantaged populations, are not unique to one context but common across countries [12]. The set of ACSC related hospitalizations as defined by AHRQ is not validated for the Portuguese population, but has been validated in Italy, a southern European country with universal coverage [26].

Second, the inequalities found in ACSC related hospitalizations may also be driven by other patient characteristics such as comorbidities and by disease prevalence. There is no regular available data on disease prevalence in Portugal, so we used as covariates the disease specific mortality rate of the three chronic conditions that contribute notably for ACSC, as was used in previous studies [5]. Regarding patient comorbidity, in contrast with other methodologies, AHRQ performs an annual revision of the diagnosis/procedure codes and has defined an extensive list of exclusion criteria for each ACSC, which allows for the adjustment for some coexisting conditions. Also, there is some evidence that other determinants of health, like the percentage of smokers and proportion of people that are physically inactive could influence the rate of ACSC related hospitalizations [40]. Again, in Portugal there is no data for these variables at the municipality level, and additionally, other studies showed that the SE inequalities remained practically unchanged after controlling for lifestyle variables [11, 16, 41]. This may be due to the fact that lifestyles are strongly associated to SE factors.

Third, in Portugal, the inpatient database does not contain any information on the SE status of the patient. Thus, we used area-based variables of the patients’ municipality of residence. Even though this could lead to ecological fallacy, because the observed correlations at the municipality level might not be true at the individual level [42], our main objective was to describe inequalities and not to measure causalities. Note that this assumption is valid and is commonly used in similar studies [12, 34, 41, 43], as well as in many other studies on the association of health outcomes and area specific SE characteristics [24, 44]. Furthermore, ecological studies have the advantage of using large existing datasets and can include a large number of people to test hypotheses.

Finally, we used the DRG tariffs as a proxy for the cost of each ACSC related hospitalization. Nevertheless, DRGs are widely used for hospital prospective payment schemes and the price of each DRG are widely used as a proxy for the costs, since is the best proxy for hospital costs of each in-patient episode [45, 46].

## Conclusion

Despite universal coverage and relatively low co-payments in PC, in Portugal there are substantial SE inequalities in ACSC related hospitalizations, possibly reflecting inequalities in early and preventive high-quality care. Furthermore, these inequalities increased from 2000 to 2014, possibly contributing to the widening of the health gap, and represent a substantial financial burden for the Portuguese NHS. These results reflect the current lack of a national-oriented research strategy on health inequalities and point to the need to implement effective public policies to reduce social inequalities. Further research should be developed to understand why individuals from low SE areas are more likely to be hospitalized for avoidable reasons, and how the quality of preventive and PC services influence these specific ACSC related hospitalizations, in order to reduce current inequalities and their associated costs.

## Abbreviations

ACSC: Ambulatory Care Sensitive Conditions
DRG: Diagnosis Related Groups
NHS: National Health Service
PC: Primary Care
PQIs: Prevention Quality Indicators
SE: Socioeconomic
